# Proteomic and functional characterisation of extracellular vesicles from collagen VI deficient human fibroblasts reveals a role in cell motility

**DOI:** 10.1038/s41598-023-41632-1

**Published:** 2023-09-05

**Authors:** Carmen Badosa, Mónica Roldán, Joaquín Fernández-Irigoyen, Enrique Santamaria, Cecilia Jimenez-Mallebrera

**Affiliations:** 1 Neuromuscular Unit, Neuropaediatrics Department, Institut de Recerca Sant Joan de Déu, Hospital Sant Joan de Déu, PCCB, 3rd Floor, Calle Santa Rosa 39-57, 08950 Barcelona, Spain; 2 Confocal Microscopy and Cellular Imaging Unit, IPER, Department of Genetic and Molecular Medicine, Institut de Recerca Sant Joan de Déu, Hospital Sant Joan de Déu, Barcelona, Spain; 3 Proteomics Platform, Proteored-ISCIII, Clinical Neuroproteomics Unit, Navarrabiomed (CHN-UPNA-idiSNA), Pamplona, Spain; 4 Center for Biomedical Research on Rare Diseases (CIBERER), Instituto de Salud Carlos III, Madrid, Spain; 5 Department of Genetics, Microbiology and Statistics, University of Barcelona, Barcelona, Spain

**Keywords:** Neuromuscular disease, Mechanisms of disease

## Abstract

Extracellular vesicles (EVs) are key mediators of cell-to-cell communication. Their content reflects the state of diseased cells representing a window into disease progression. Collagen-VI Related Muscular Dystrophy (COL6-RD) is a multi-systemic disease involving different cell types. The role of EVs in this disease has not been explored. We compared by quantitative proteomics the protein cargo of EVs released from fibroblasts from patients with COL6-RD and controls. Isolated EVs contained a significant proportion of the most frequently reported proteins in EVs according to Exocarta and Vesiclepedia. We identified 67 differentially abundant proteins associated with vesicle transport and exocytosis, actin remodelling and the cytoskeleton, hemostasis and oxidative stress. Treatment of control fibroblasts with EVs from either patient or healthy fibroblasts altered significantly the motility of cells on a cell migration assay highlighting the functional relevance of EVs. In parallel, we analysed the secretome from the same cells and found a distinctly different set of 48 differentially abundant proteins related to extracellular matrix organisation and remodelling, growth factor response, RNA metabolism and the proteasome. The EVs and secretome sets of proteins only shared two identifiers indicating that the sorting of proteins towards EVs or the secretory pathway is tightly regulated for different functions.

## Introduction

Cell-to-cell communication is necessary to maintain tissue homeostasis in physiological conditions through the delivery of mediators counted for hundreds, between different cell types. Part of this exchange takes place through the release of molecules directly to the extracellular space or by direct contact between cells by means for example of gap junctions and nanotubules depending on the setting. Additionally, extracellular vesicles (EVs) mediate short- and long-range exchange of molecules between cells although their physiological role is still not completely understood. The release of EVs is an evolutionarily conserved mechanism from microorganisms to mammals. Furthermore, the content of Evs released from diseased cells is thought to reflect the pathological state of that cell or to represent unwanted components that need to be discharged. In this sense, studying EVs offers a means to understand disease progression and to identify specific disease molecular biomarkers with the added value that those are protected by the EVs membranes and therefore more stable than free circulating molecules^1,2^.

Collagen VI-related muscular dystrophy (COL6-RD) is an example of a multi-systemic disease involving different cell types^3^. The collagen VI tetramers are synthesised and secreted by fibroblasts present in the interstitial space in each corresponding tissue. In skeletal muscle, collagen VI is released by endomysial fibroblasts and once in the extracellular matrix interacts with other extracellular proteins and components of the muscle basement membrane connecting at the structural and signaling levels the muscle cell with the surrounding connective tissue^4^. We and others have described that, mutations in collagen VI genes leading to collagen VI defects result in a global phenotypic change of the fibroblasts which is reflected in very significant changes in their gene expression profile and specific functions such as adhesion^5^ as well as organelles structure organization^6^.

Here we aimed to characterise the protein content of EVs released from primary skin fibroblasts of patients with collagen VI deficiency, as a disease cell model, and compare it with the set of proteins secreted directly to the medium by the same cells (secretome) as a first step towards understanding the contribution of EVs mediated cell communication in this disease and to search for specific biomarkers.

Proteomic analysis revealed that they contained known markers of exosomes such as CD81 and flotillin-1 and a significant proportion of the top 100 most frequently reported proteins in exosomes and EVs according to Exocarta and Vesiclepedia^7,8^. They also contained proteins in common with other reported studies of exosomes and other EVs in fibroblasts^9–11^. We identified by label-free quantitative proteomics a set of 67 differentially abundant proteins between patients and control fibroblasts derived from EVs of which only three were common to the set of differentially abundant proteins in the secretome. EVs-specific proteins were functionally associated with hemostasis, vesicle transport and exocytosis, actin remodelling and the cytoskeleton, cell adhesion, brain development and oxidative stress. Furthermore, we searched for candidate proteins in the resulting comparisons for potential biomarkers and found MMP-3 significantly increased in serum from COL6-RD patients.

At the functional level, we found that patient-derived EVs had a specific effect on control cell motility, altering their speed and their directionality in a cell wound migration assay.

In summary, this study is the first description of EVs in the context of congenital muscular dystrophies and shows that collagen VI deficient fibroblasts pack and release in a directed manner a set of specific proteins which are likely to reflect their pathological state and may have an impact on neighboring cells ‘function serving thus as mediators of disease progression. Further studies are required to evaluate the relevance and application of such findings in this disease, perhaps to monitor individual disease progression or future therapies.

## Results

**Figure 1:**
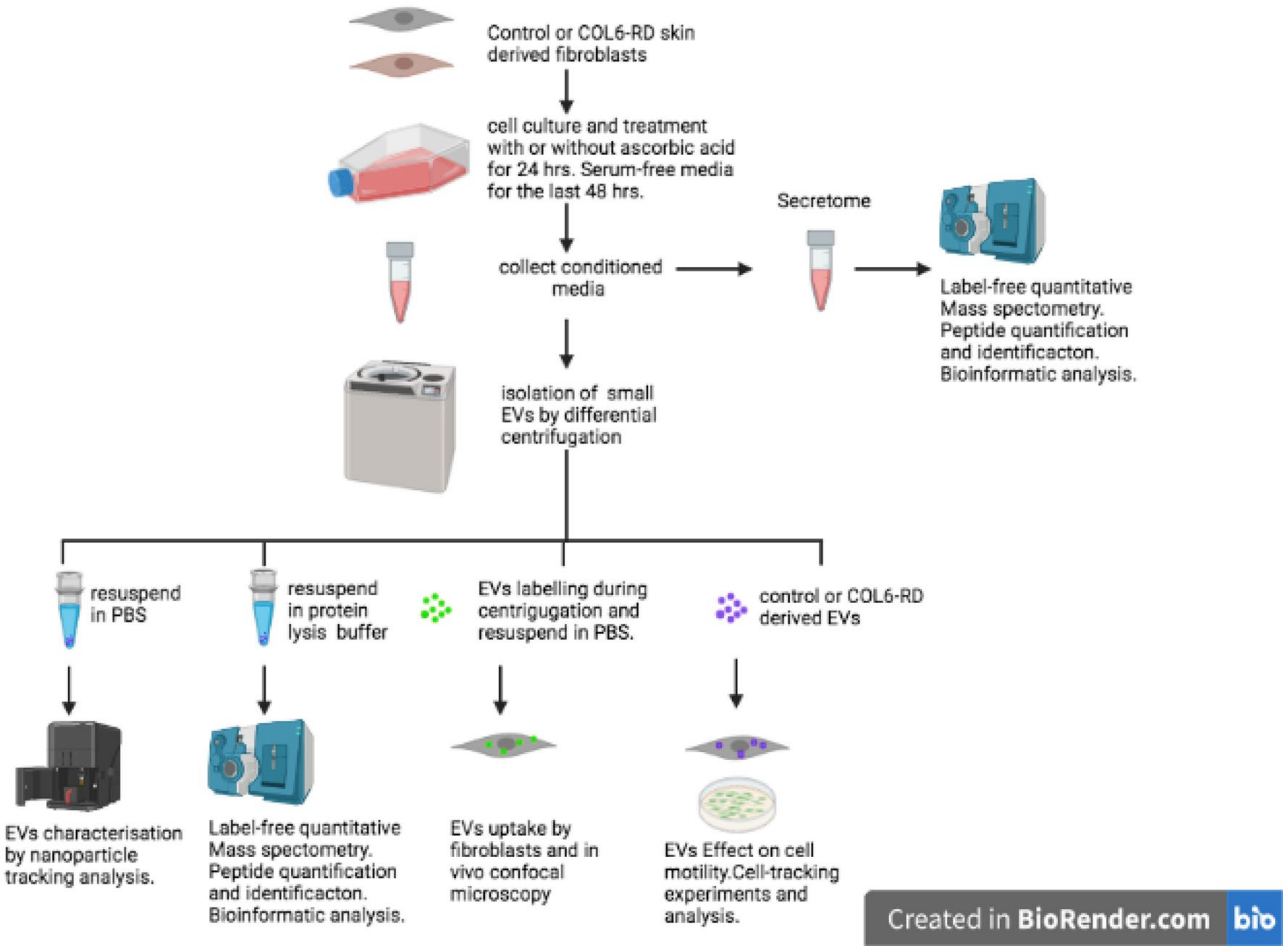
Experimental workflow.

The experimental workflow is summarised in Fig. 1

### Characterisation of isolated particles

Differential ultracentrifugation is one of the most used primary EV separation and concentration techniques. This is regarded as an intermediate recovery and intermediate specificity separation method which yields a mix of EVs and a limited amount of other non-EV components. There is no one-size-fits-all approach to isolate EVs, and we believed that this method was appropriate for an overall description of the contribution of the extracellular compartment to collagen VI deficiency and because the focus was on the comparison between patients and control cells derived EVs isolated in parallel and under the same conditions. Moreover, ideally, any valuable biomarker identified this way should be measurable in samples that require as little preparation as possible so that it is clinically relevant. Regarding functionality, given that the role of individual EV populations is not fully understood and considering that they may act synergistically there is value in considering the pool of EVs as a whole-cell compartment as discussed previously^2^.

Based on the particle nano tracking analysis (NTA) performed, the mean size of the particles in a representative sample (n = 3 measurements) was 220 nm ± 1.5 nm and the mode was 172 nm ± 11.4 nm. The mean concentration was 1 × 10^9^ particles/mL (54.5 ± 3.0 particles/frame). Thus, the total amount of EVs in each sample was on average 1 × 10^8^. Also, based on NTA, the fraction of particles within the exosomes range (35–155 nm) was 30% of the total of particles whereas the majority of particles (70%) were larger than 155 nm and smaller than 500 nm. Particles below 35 nm or above 500 nm represented less than 2% of the total. These results are similar to others reported in similar studies using ultracentrifugation to isolate EVs from human fibroblasts^12,13^. Hereafter we will apply the generic term extracellular vesicle (EV) to refer to this specific population of particles.

### Proteomic analysis of EVs and functional enrichment

Since we were performing proteomic analysis of the content of EVs, the data from one of the control samples served to confirm the enrichment of the preparations in markers of exosomes and other EVs. Our proteomic approach identified 862 proteins, 445 of those with at least two unique peptides (Supp. Table 1). The mass spectrometry raw data and search results files have been deposited to the ProteomeXchange Consortium (http://proteomecentral.proteomexchange.org) via the PRIDE partner repository^14^ with the dataset identifiers PXD018765 (https://repository.jpostdb.org/preview/42145518764a55ad7afa21 ; Access key 1100)^15^. As shown in Supp Fig. 1, EV-associated proteomes and secretomes derived from collagen VI deficient fibroblasts presented more heterogeneity than the control group.

**Figure 2:**
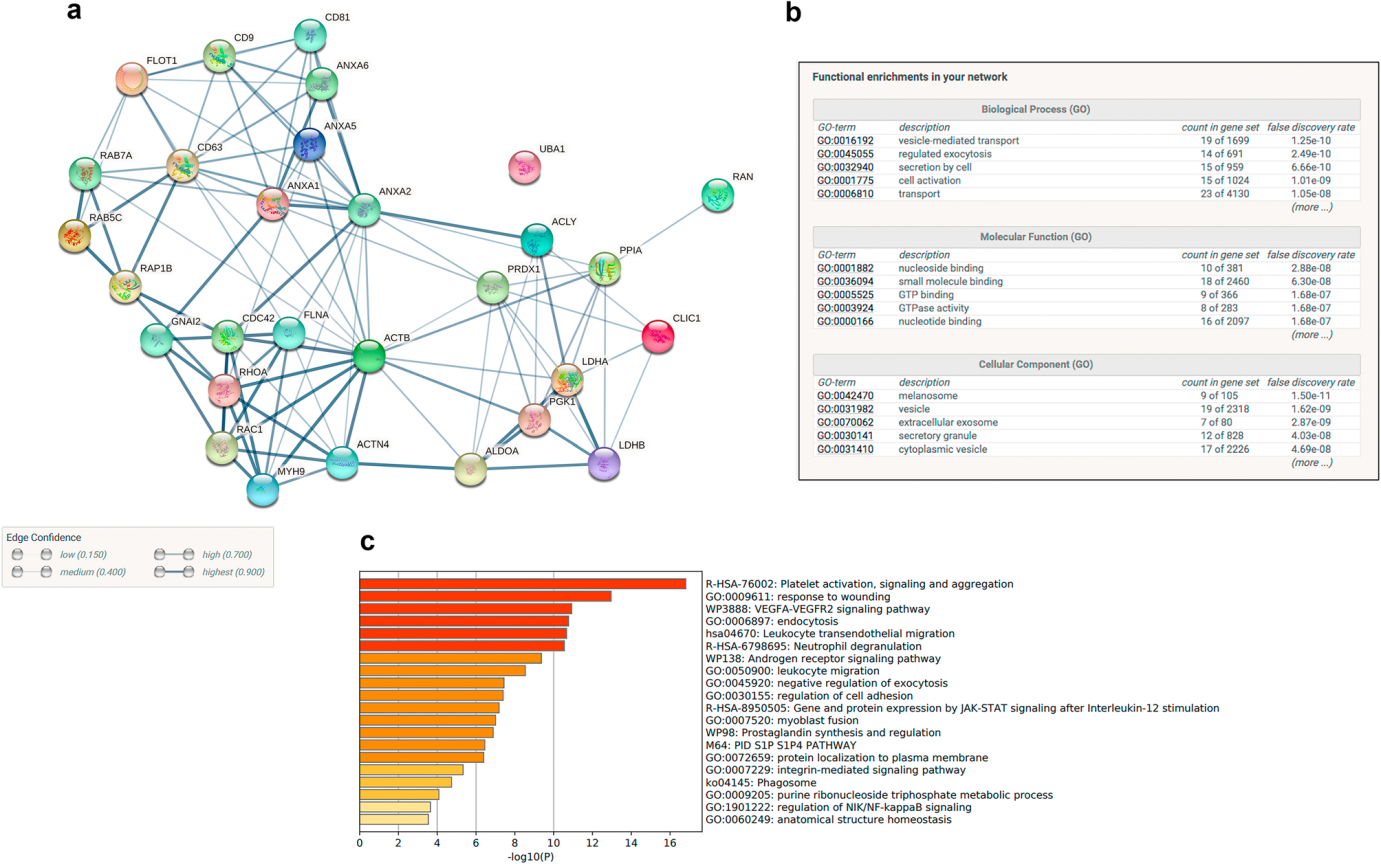
STRING protein interaction network of the common proteins in EVs for our study and the top 100 EVs proteins according to Vesiclepedia and Exocarta (**a**). Edges represent interactions based on confidence level. Functional enrichment of this set of proteins based on Gene ontologies (**b**) and the top 20 functional categories (**c**) according to Metaescape (*p* < 0.05) are shown.

Of those 862 proteins, 29 were common with the top 100 proteins reported in exosomes and EVs according to two databases (Exocarta and Vesiclepedia, Supp. Table 1. This group of 29 proteins were highly interconnected as shown by STRING analysis^16^, (Fig. 2, gene symbols are shown). The connections (edges) between the proteins (nodes) indicate the confidence level of the data supporting those interactions in the shown network. One of the protein clusters included surface receptors that are considered as EVs markers such as CD63, CD81, CD9 and Flotillin-1 which are involved in caveolae formation. Another cluster contained several annexin family members, one group of proteins was involved in actin and myosin cytoskeleton, vesicular trafficking and signal transduction whilst another one in glucose metabolism. The most represented gene ontologies amongst those 29 components in terms of Biological Process were vesicle-mediated transport, regulated exocytosis, secretion by cell, cell activation and transport and in terms of cellular compartment the melanosome, vesicle, exosome, secretory granule and cytoplasmic vesicle (Fig. 2b) confirming the enrichment of the preparations in EVs. Figure 2c shows the top 20 functional categories according to Metascape.

Amongst the 445 proteins with at least two peptides, we found more than one representative of each category for the characterisation of EVs according to the MISEV2018 guidelines^17^. These included transmembrane or GPI-anchored proteins such as tetraspanins (CD63, CD81), MHC Class I (HLA-A), integrins (several ITGA chains), LAMP1 proteoglycans and complement binding proteins (CD59). Regarding category 2 of cytosolic proteins, we detected Flotillin-1, annexins, actin and tubulin as well as secreted proteins recovered with EVs including adhesion and extracellular matrix proteins (collagen alpha chains, MFGE8 and other extracellular proteins). We also identified some proteins that are major constituents of non-EV structures including apolipoprotein and albumin and proteins of non-EV subcellular compartments such as pre-lamin A/C although it cannot be completely ruled out that some of those markers may be also associated with some EVs. Therefore, these data suggest that the population of vesicles that we isolated are mainly EVs with limited non-EVs components.

**Figure 3:**
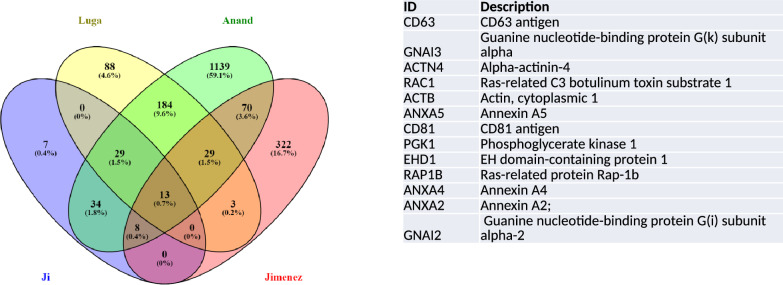
Venn´s diagrams representing common proteins between existing datasets (from Vesiclepedia) of EVs from mouse fibroblasts and the 445 EV derived proteins identified in the present study.

We searched Vesiclepedia for other datasets of fibroblasts. We found three studies that were conducted on mouse fibroblasts but none on human fibroblasts^9–11^. We compared those three data sets (Ji, Anand and Luga) with our study (Jimenez) using the gene symbols provided in Vesiclepedia as IDs and Venny tool^18^. Amongst the 445 IDs identified by us with more than 2 peptides, there were 21 gene symbols in common with the 91 reported by Ji et al., (23%); 45 out of 346 gene symbols in common for Luga et al., (13%) and 120 gene symbols in common between our study and Anand et al., (8%)^9–11^. The four studies shared 13 gene symbols (Fig. 3 and Supp. Table 1). The corresponding proteins were associated with the cell surface (Annexins A2, A4 and A5), actin cytoskeleton (alpha-actinin, actinin-cytoplasmic) vesicle trafficking (CD63, CD81, Ras Family Small GTP Binding Protein RAP1B, Rac1 and EH Domain containing protein 1, EHD1), metabolism (Phosphoglycerate kinase 1, PGK1) and signal transduction (G protein subunits I2 and I3, GNAI2 and GNAI3 and Rac Family Small GTPase 1) (Fig. 3). These could therefore represent useful markers of EVs specifically derived from fibroblasts.

**Figure 4:**
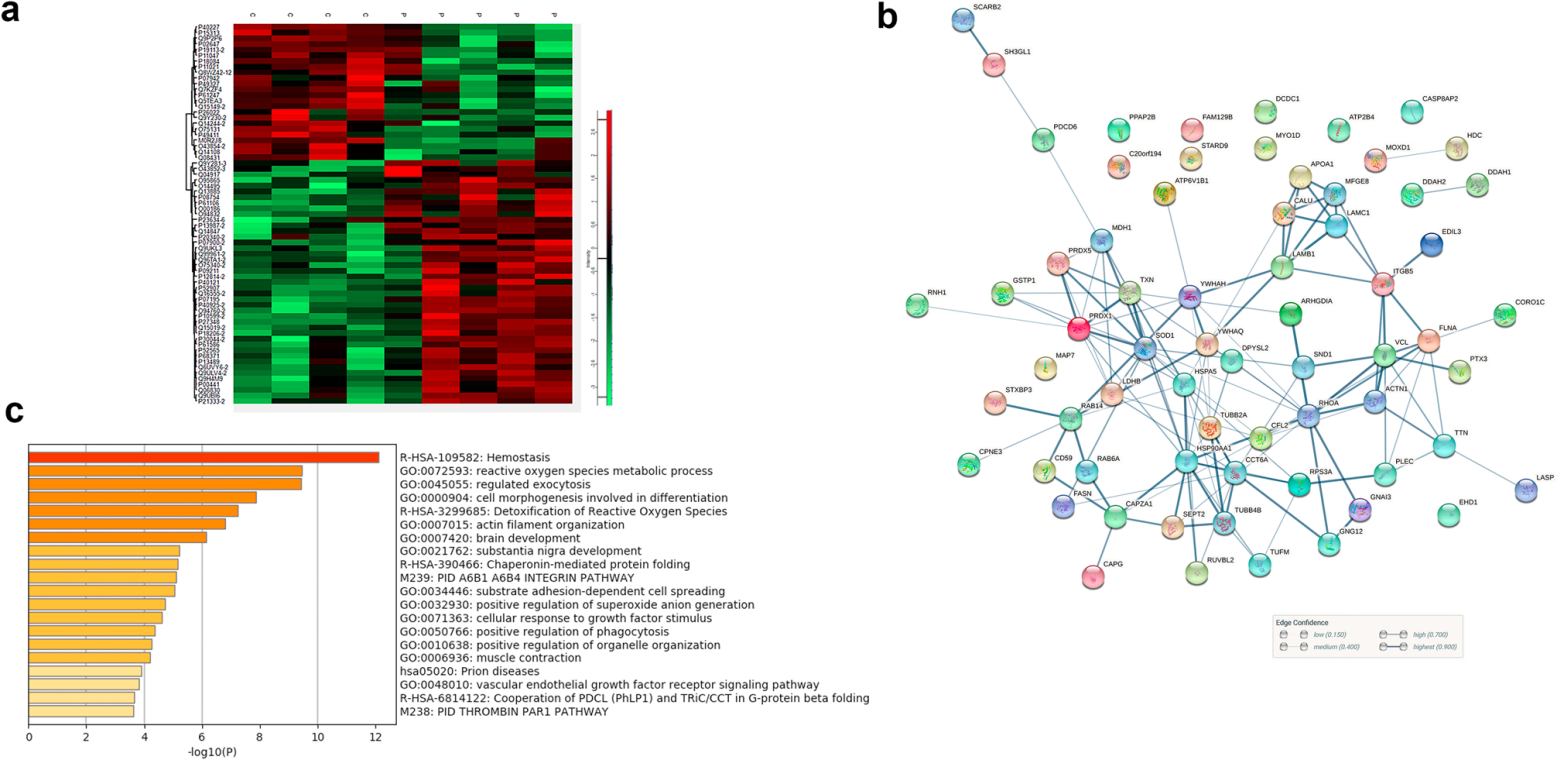
(**a**) Heatmap showing the expression levels of the DA proteins across control and patient samples. (**b**) Protein–protein interaction network (STRING) of the 67 differential proteins between COL6-RD fibroblasts (edges represent interactions based on confidence level). (**c**) functional enrichment showing the top 20 categories (based on Metaescape, *p* < 0.05).

Once we have determined that we had enriched for EVs we compared the set of proteins identified in each sample as follows: control fibroblasts (C) vs patient fibroblasts (P) and control fibroblasts treated with ascorbic acid (CAA) vs patient fibroblasts treated with ascorbic acid (PAA). The number of differential proteins between control and patient samples was higher in the cells treated with AA (140 proteins) than in untreated cells (24 proteins). For this reason, we decided to consider for further analysis the proteins with differential abundance obtained using the patient and control cells all together with and without AA and obtained 140 unique proteins. From those, we excluded those with only one peptide and the keratins which we considered as non-specific contamination, and we obtained a list of 67 differentially abundant (DA) proteins (Supp Table 1, Supp Fig. 1). Differential heatmap and the STRING protein interaction network of these 67 proteins are shown in Fig. 4a and b. Estimations performed by STRING indicate that this network has a higher number of interactions or edges (129) than expected (55) for a random set of proteins of similar size, drawn from the genome which means that this group of proteins are biologically connected (PPI enrichment *p*-value: < 1.0e−16, protein–protein-interaction enrichment). Protein annotations for these proteins is provided in Supp. Table 1.

Metaescape^19^ was used for gene annotation analysis and visualisation (based on GO, KEGG pathways, Reactome and GeneSet Enrichment analysis). The top 20 enriched ontology clusters are shown in Fig. 4c. They were mainly associated with hemostasis, reactive oxygen species, cell morphogenesis, actin filament organisation, exocytosis, phagocytosis and organelle distribution, brain development and response to growth factor stimulus, chaperonin-mediated protein folding and prion disease, integrin pathway and substrate adhesion-dependent cell spreading and most proteins were localised to the cytoplasm, cytoplasmic vesicle and the cytoskeleton. It is worth highlighting that 8 out of these 20 over-presented functional categories are associated with components of the cytoskeleton (actin and other cytoskeletal proteins such as plectin and filamin) and its remodelling during different processes (including exocytosis, cell morphogenesis, adhesion, and cell spreading).

If we focus on the extent of the fold change amongst those 67 differentially abundant proteins, we found quite important changes for proteomic analysis (Supp. Table 1). The cytoplasmic form of malate dehydrogenase was the most differentially abundant protein in COL6-RD cells (fold change, FC, 10.8×) which is responsible for the oxidation of malate to oxaloacetate in the citric acid cycle. This was followed by Ras-related protein 14 (FC 10.4×) which belongs to the RAB family of GTPases involved in intracellular membrane trafficking including Golgi to endosome transport of FGFR-containing vesicles and cell–cell adhesion. In the third position, the Phospholipid phosphatase 3 protein (FC 8.8 x) encoded by the *PLPP3* gene is a membrane glycoprotein localized at the cell plasma membrane. It functions in de novo synthesis of glycerolipids as well as in receptor-activated signal transduction mediated by phospholipase D and may be involved in cell adhesion and cell–cell interactions. On the other hand, the proteins that were less abundant in COL6-RD fibroblasts correspond to doublecortin domain-containing protein 1 (*DCDC1* gene, FC 0.04× or fold decrease of 25) which is a microtubule-binding protein which plays an important role in mediating dynein-dependent transport of RAB8A-positive vesicles, EGF-Like Repeat And Discoidin I-Like Domain-Containing Protein 3 (EDIL3 gene, FC 0.15×, fold decrease of 6.6) which is an integrin ligand that plays an important role in mediating angiogenesis and may be important in vessel wall remodelling and development and Apolipoprotein A-I (*APOA1* gene, FC 0.38×, fold decrease of 2.6) which promotes cholesterol efflux from tissues to the liver for excretion (source Gene cards https://www.genecards.org/).

### Proteomic analysis of the secretome and functional enrichment

According to the Human Protein Atlas, a secretory protein can be defined as a protein which is actively transported out of the cell. In humans, cells such as endocrine cells and B-lymphocytes are specialized in the secretion of proteins, but all cells in the body secrete proteins to varying degrees. Proteins that are secreted from the cell play a crucial role in many physiological, developmental and pathological processes and are important for both intercellular and intracellular communication. In addition to being a rich source of new therapeutics and drug targets, a large proportion of clinically relevant blood diagnostic tests are directed towards secreted proteins, emphasizing the importance of this class of proteins for biomedicine.

To compare the proteins preferentially packed into EVs to those secreted directly to the extracellular space we analysed in parallel and using the same label-free proteomic approach the secretome of the same COL6-RD and control cells. We quantified 432 proteins with at least 2 unique peptides (Supp. Table 1) and obtained 48 differentially abundant proteins with two or more unique peptides between patients and controls (Supp. Table 1).

**Figure 5:**
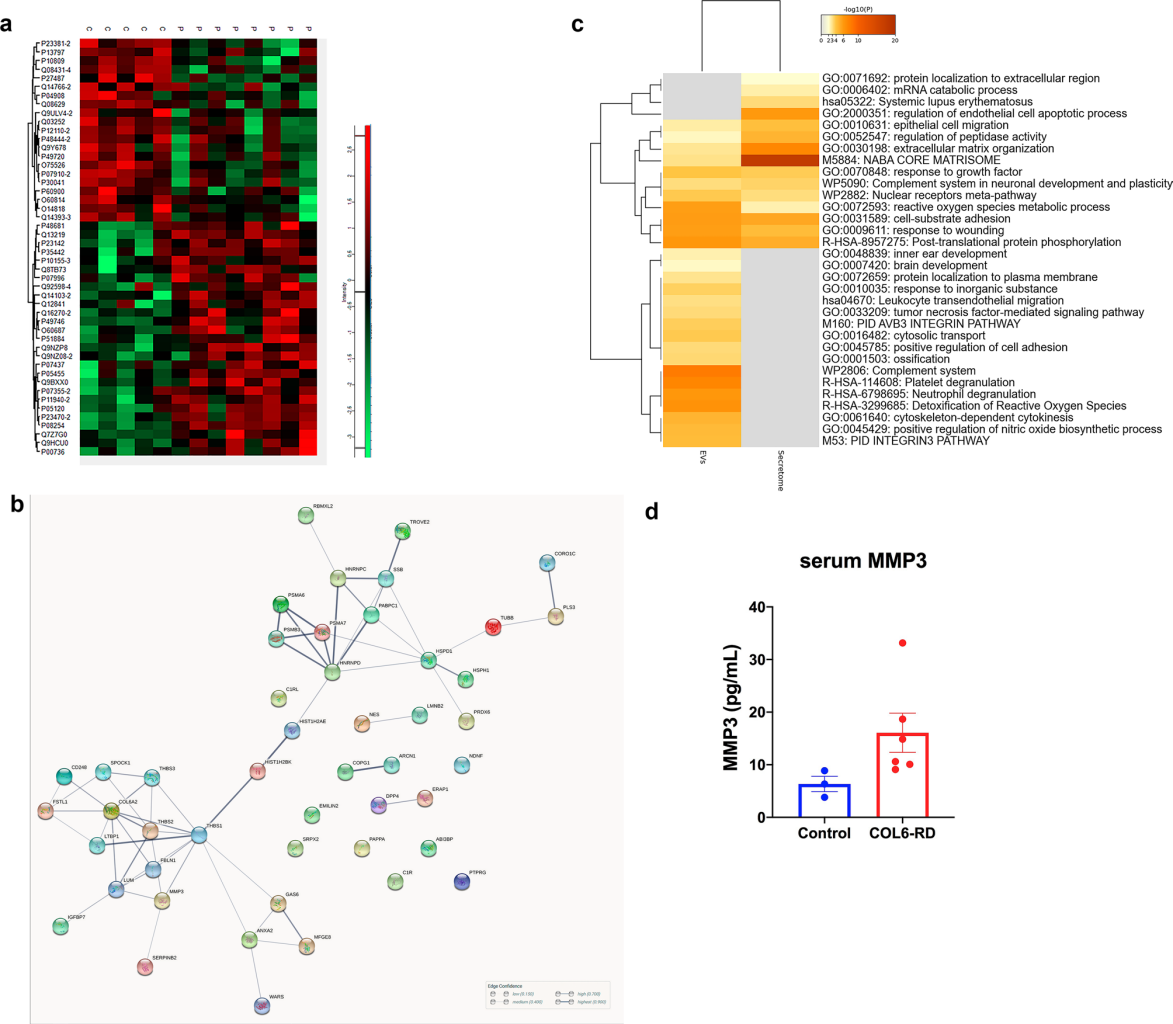
(**a**) Heatmap showing the expression levels of the DA proteins across control and patient samples. (**b**) Analysis of the secretome showing the protein–protein network of the 48 differentially abundant proteins in COL6-RD derived fibroblasts versus healthy fibroblasts. (**c**) Comparative meta analysis of the most overrepresented functional categories amongst the DA proteins in EVs versus those in the secretome (Metaescape). (**d**) Results of the MMP3 ELISA showing an increased concentration in serum from COL6-RD serum (mean values and SEM).

The differential heatmap corresponding to the differential protein set detected at the secretome level is shown in Fig. 5a. STRING protein network analysis revealed two tightly associated clusters among these 48 proteins; one associated with the extracellular matrix which included thrombospondins, collagen, MMP3, proteoglycans and growth factors, and a second cluster composed of various components of the proteasome, heat shock proteins, annexins and tubules (Fig. 5b). For this network as a whole, the PPI enrichment *p*-value was < 1.0e−16 since more interactions than expected were determined. Meta-analysis of enriched functions and pathways represented amongst the DA proteins in EVs and those in the secretome showed very little overlap apart from some functions related to cell adhesion (Fig. 5c).

For example, DA abundant proteins in the EV dataset were related to the response of granulocytes and platelets to infection and vascular injury respectively, via the release of granules, whereas these functions are not represented in the set of DA proteins in the secretome. This is also the case for certain cell signalling pathways such as the Integrin 3 cell surface interaction according to the protein interaction database the reactive oxygen species detoxification and nitric oxide synthesis. In contrast, certain functions and gene ontologies such as extracellular matrix organization and the matrisome where much more over-represented in the set of DA secretome proteins than in the EVs dataset.

Furthermore, when we compared the set of 67 DA proteins in EVs with the 48 DE proteins identified in the secretome and only two proteins were shared (Lactadherin, gene *MFGE8* and Coronin-1C, *CORO1C)*. Thus, this confirms that the EVs and the secretome constitute two very well-differentiated cell compartments and that secretion into the extracellular space of both sets of proteins is a highly regulated process.

### Search for secreted proteins and candidates for circulating biomarkers

One of the objectives of this study was to identify candidate circulating biomarkers for COL6-RD that could be readily detected in biological fluids. To this aim, we selected a few candidates amongst the differentially abundant proteins in fibroblasts from COL6-RD patients..

To inform this selection, we searched databases of secreted proteins, which a priori should be accesible in biological fluids. Proteins are mainly secreted into the extracellular space through the classical secretory pathway. These proteins contain a hydrophobic signal-peptide sequence, which mediates their translocation to the ER/Golgi and their release from the cell by exocytosis^20^. We crossed our list of DA proteins in the secretome with a catalogue of 1539 human protein-coding genes predicted to encode secreted proteins^20^ and identified 8 proteins in common (Supp. Table 1).

These 8 proteins represent good candidates for circulating biomarkers. Amongst those, we chose the two top proteins with the highest FC in the comparison between patient and control cells (Matrix-Metalloproteinase 3, MMP3, and Neuron Derived Neurotrophic FactorNDNF). In addition, we also decided to look at Malatae Dehydrogenase 1, MDH1, which was the protein with the highest FC in the comparison between patient and control EVs. We looked at the circulating concentration of these three proteins using commercially available ELISA kits. Serum MMP3 was significantly elevated (*p* = 0.02, Mann–Whitney) (using a commercially available ELISA) in patients with confirmed mutations in collagen VI (n = 6) including three patients that were not part of the initial proteomic screening (Fig. 5d) and relative to aged, matched controls (n = 3). We did not obtain differences for NDFN or MDH1 (data not shown).

### Cell motility tracking experiments

Given that the differentially abundant proteins between healthy and COL6-RD derived EVs was related to the remodelling of the actin cytoskeleton and substrate-dependent cell spreading, according to the functional enrichment analysis, we hypothesised that when these EVs reach their target cell they may exert an effect on their motility. Therefore, we designed a set of experiments to investigate if the proteins identified in control and COL6-RD EVs were functionally active in participating in cell migration by applying a wound assay. For this reason, we focused on comparing the effect of EVs isolated from either healthy or COL-6 RD EV on the migration of healthy cells. The basal condition in this case corresponds to the untreated healthy cells and the healthy cells treated with EVs from controls.

Firstly, we analysed the ability of isolated EVs to enter fibroblasts in culture and their distribution by marking EVs with DIO and performing in vivo confocal microscopy (Supp. Video 1). Control cells were grown with labelled EVs from their own extracellular medium in a medium depleted from EVs. After 24 h of treatment, we could see that labelled EVs had entered virtually all cells and that their abundance amongst cells was uniform albeit with slight differences.

To investigate the effect of EVs on cell motility we performed a migration assay based on the wound healing method. We used 35 mm plates that contained a 2-well silicone insert separated by a 500um cell-free gap that represented the wound (see Materials and Methods). First, we determined the time and conditions required by primary fibroblasts to close the wound using a control fibroblast cell line not treated with EVs. Untreated cells required approximately 24 h to completely close the wound (data not shown). The addition of the cell tracker marker used for in vivo confocal microscopy did not affect cell migration. Afterwards, we established the conditions for healthy fibroblasts treated with EVs from healthy fibroblasts and found similar results therefore we adopted those conditions for all the in vivo imaging experiments. Given that we wanted to analyse the motility of individual cells before the closure of the wound, we chose a timeframe of 17 h (overnight) for the analysis.

**Figure 6:**
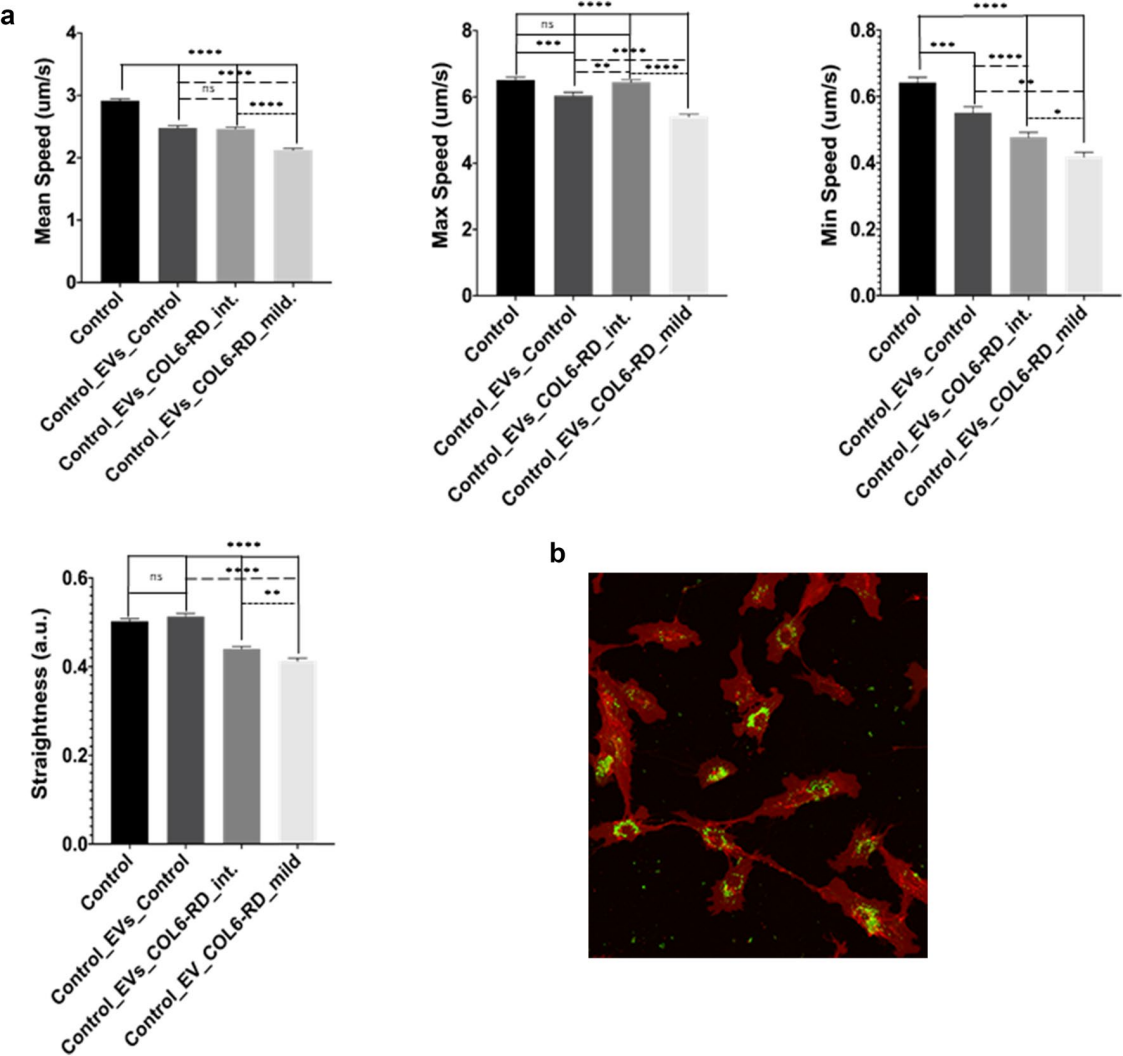
(**a**) Results of cell tracking assay showing the mean, maximum (max) and minimum (min) speed of control cells either untreated or treated with EVs derived from control fibroblasts, EVs derived from a COL6-RD muscular dystrophy patient with an intermediate phenotype and a COL6-RD muscular dystrophy patient with a mild phenotype. Continuous lines represent comparisons with untreated control fibroblasts, dashed lines with control cells treated with EVs derived from control fibroblasts and dotted lines between control cells treated with EVs from either mild or intermediate COL6-RD fibroblasts. * *p* < 0.05, ** *p* < 0.01, *** *p* < 0.001, **** *p* < 0.0001 (**b**) Representative image of labelled EV´s incorporation in control fibroblasts in vivo.

We observed cells moving towards the wound in all conditions (Supp. Video 2–5). The movement of untreated cells and cells treated with EVs from the same control cell line appeared quite similar. However, in the cells treated with EVs from either patient, we observed that instead of moving with a clear direction towards the other side of the wound they moved in different directions, returning in some cases towards their origin. Also, cells from the patient with the milder phenotype moved more slowly than the other three cell lines. These observations were confirmed when we performed the detailed image analysis in particular with regards to the mean and minimum speed and the cell directionality represented by the measurement of the trajectory straightness (Fig. 6). We found significant differences for these three parameters between conditions (one-way ANOVA, *p* < 0,0001, for all three). When performing ad-hoc comparisons between two conditions only, we found significant differences in all cases except for the comparison between control cells treated with EVs from control cells and EVs from the COL6-RD patient with an intermediate phenotype (Tukey's multiple comparison test, Table 1).

**Table 1:** Results of Tukey´s multiple comparison test (adjusted *p*-value for the means) for the different comparisons and the selected cell speed and trajectory parameters.

Parameter	Control versus Control_EVs_Control	Control versus Control_EVs_COL6-RD_int	Control versus Control_EVs_COL6-RD_mild	Control_EVs_ Control versus Control_EVs_COL6-RD_int	Control_EVs_ Control versus Control_EVs_COL6-RD_mild	Control_EVs_COL6-RD_int_Control_EVsCOL6-RD_mild
Mean speed (um/s)	*p* < 0.0001	*p* < 0.0001	*p* < 0.0001	ns	*p* < 0.0001	*p* < 0.0001
Max speed (um/s)	*p* < 0.001	ns	*p* < 0.0001	*P* < 0.01	*p* < 0.0001	*p* < 0.0001
Min speed (um/s)	*P* < 0.001	*p* < 0.0001	*p* < 0.0001	*P* < 0.01	*p* < 0.0001	*P* < 0.05
Straightness	Ns	*p* < 0.0001	*p* < 0.0001	*p* < 0.0001	*p* < 0.0001	*P* < 0.01

## Discussion

Proteomics and other high throughput approaches provide an unbiased birds-eye view of a particular disease from which to focus on more specific aspects and pathways based on the initial results. The current study represents the first proteomic description of the cargo of extracellular vesicles (EVs) and the secretome in fibroblasts from patients with collagen VI-related muscular dystrophy (COL6-RD), completing our previous transcriptomic studies and the work of other groups^5^ towards a comprehensive description of the main cell target (fibroblast) and the most relevant cellular localisation (the extracellular space) in this condition. In addition to furthering our understanding of COL6-RD, this knowledge could help identify biomarkers and ligands for the functionalization of vehicles (such as lipid nanoparticles) for targeted therapeutic delivery amongst other applications.

The investigation of EVs in the context of neuromuscular diseases and muscular dystrophies is a yet relatively unexplored field. Several studies have been published for the most prevalent or more widely known neuromuscular conditions including Amyotrophic Lateral Sclerosis (ALS), Spinal Muscular Atrophy (SMA), Duchenne Muscular Dystrophy (DMD) and inflammatory myopathies. In the latter, there are indications that EVs promote muscle inflammation and weakness in polymyositis and dermatomyositis. EVs derived from inflammatory cells and platelets are elevated in plasma from patients with inflammatory myopathies and EVs from plasma from patients with juvenile dermatomyositis are taken up by aortic endothelial cells where they modified their gene expression profile^14,21^.

ALS is probably where more literature is available. A comparison between plasma EV from a large cohort of ALS patients and other neurodegenerative diseases and controls showed that EVs contained known markers of ALS such as HSP90, PPIA or cyclophilin A and TDP-43. Moreover, mathematical models combining, the size distribution of EVs and the abundance of HSP90 gave excellent results when distinguishing ALS from the other pathological groups and healthy controls^22^. Therefore, the different features of EVs may help towards better classification and prediction of patients. Interestingly, in our dataset HSP90 was slightly increased in EVs from patients´ cells and PPIA was one of the 29 proteins in common between our dataset and the Top 100 reported proteins in EVs and exosomes.

The direct comparison between the proteomic signature of EVs and directly secreted proteins from the same cells allows us to conclude that both secretory pathways are completely independent in origin and function and that there is no accidental release of proteins from EVs into the extracellular medium. Whilst the protein content of fibroblast-derived EVs was enriched in intracellular and cytoskeletal proteins, their secretome was enriched in extracellular matrix proteins some of which correspond to genes that we have found differentially expressed in our previous transcriptomic studies of fibroblasts and muscle tissue from COL6-RD patients^5,23^. This is the case for MMP3 which is a metalloproteinase involved in ECM remodelling and together with MMP9 is one of the recurrent candidates for serum biomarkers for other muscular dystrophies^24^.

The wide range of the functional effects of EVs on the target cell is starting to emerge, largely from studies in the field of tumour biology. Cell migration is critical for homeostasis in pluricellular organisms and depends on different extracellular cues including chemical and mechanical signals. Genetic ablation of exosome secretion in cancer cells greatly affects not only the speed of migration but also their directionality^25^. In the present study, we also found that treatment of healthy fibroblasts with EVs secreted by collagen VI deficient fibroblasts resulted in significant changes in speed and directionality as determined by our quantitative image analysis in a migration assay.

We postulate that that this effect may be mediated by proteins which are contained within EVs and involved in cell motility. To elucidate which of the proteins present in EVs could play a role in cell migration as determined by the wound assay results we searched amongst the differentially abundant proteins for candidates described in the literature as being involved in actin-cytoskeleton remodelling as well as in EVs biology. We identified the Small GTP Binding Protein RAB14, RAB14, as one possible link between the effect of COL6-RD-derived EVs on cell motility and the results of the proteomic analysis. This is a small GTPase related to lamellipodia formation that is considered an oncogene since it is over-expressed in different types of tumours. It interacts with the late endosome and exosome marker CD63 and it has been shown to regulate exosome biogenesis and maturation. It is a downstream target of Nischarin which is a tumour suppressor. In breast cancer-derived cells, over-expression of Nischaring inhibits RAB14 activity reducing exosome shedding and spreading on exosomes^26^. In our system, EVs from COL6-RD transport an increased amount of RAB14 which on entering the target cell (fibroblasts) could induce changes in the phosphorylation of paxillin, and consequently in motility. Also, altered levels of RAB14 could induce changes in the number and size of released EVs in neighbouring fibroblasts. We did not observe an obvious difference in the concentration and size range of exosomes between control and COL6-RD fibroblasts on the nano tracking analysis but a more detailed analysis could be conducted in the future.

Another potential link between collagen VI deficiency and the effect of EVs on cell motility is hinted at by our recent work which demonstrates a direct functional link between collagen VI, the Capillary Morphogenesis Gene 2 Protein, CMG2, receptor and the endocytic pathway. We showed an increased number of endosomes in fibroblasts from patients with COL6-RD which was associated with increased phosphorylation of CMG2 which in turn modulates the actin cytoskeleton via RhoA and talins^6^ although this would require further signalling studies.

One of the main challenges in EVs research is the isolation of unique EVs populations characterised by different sizes, composition and cargo. Although the consecutive combination of different methods can improve their separation none of the current methods is completely infallible^27^. On the other hand, different subtypes of EVs co-exist in the extracellular space where they may act synergistically. Thus, in this study, we considered that the characterisation of the whole range of EVs was appropriate as a first step towards the study of the role of EVs in collagen VI deficiency. In future studies, we could apply other technologies that allow for example single-vesicle analysis^28^. Perhaps these highly precise studies could be more easily conducted in animal models where it is possible to obtain a larger set of tissues and derive different cell types such as fibroblasts from skeletal muscles and other connective tissues affected in COL6-RD. In this respect, we have generated a novel knock-in mouse model with a common dominant negative mutation in COL6A1 where once characterized we could analyse EVs biology and replicate some of the studies presented here.

To conclude, in this study we show that EVs from control and COL6-RD-derived fibroblasts contain a distinct set of proteins which is distinctively different from that of freely secreted proteins. An additional contribution of our findings is that EVs can modulate fibroblast motility. This represents a potential link between collagen VI deficiency and some of its clinical hallmarks such as impaired wound healing which partly depends on cell migration. Furthermore, this finding could be used as a functional "outcome measure" to monitor the efficiency of gene-modifying therapies that we and other groups are developing for these conditions^29^.

## Materials and methods

### Ethical statement

The study was approved by the Hospital Sant Joan de Déu Ethical and Scientific Committees (Ref PIC 143-16) and follows the principles of the declaration of Helsinki. All samples were provided by the Biobank of the Hospital Sant Joan de Déu. Informed consent was obtained from all study participants'.

### Patients and samples

Patients (n = 5, 3 males and two females, age range at the time of biopsy were 3–9 years) with a frequent mutation (c.877 G > A; p. Gly 293Arg) in *COL6A1* and a partial collagen VI deficiency were selected for the initial proteomics screening (4 with an intermediate phenotype and 1 with Bethlem Myopathy mild phenotype)^3^. Skin biopsies from patients were obtained from the forearm using a 4 mm punch and processed to obtain primary fibroblast cultures as we previously described^5^. Age and sex-matched control samples (n = 4) were provided by the Biobank of the Hospital Sant Joan de Déu.

### Cell culture and processing

Fibroblasts from each patient and controls were seeded (1.5 × 10^6^ cells) and cultured in 175 cm^2^ flasks in DMEM (Gibco, USA) with 10% of fetal bovine serum (FBS) (Gibco), 1X penicillin/streptomycin (Gibco), and 1X glutamine (Gibco) at 37 °C in 5% CO_2_ changing the medium (50 mL) every two days. Two flasks were used to isolate EVs and two to obtain the cell's secretome (a set of proteins freely secreted into the extracellular space). When cells were at 90% confluence (after 5 days), ascorbic acid was added at a 25ug/mL concentration for 24 h to one of the flasks for either EVs isolation or secretome collection. In all cases, cells were grown for the last 48 h in FBS free medium to avoid serum protein contamination. In addition, a protease inhibitor cocktail was added to the medium. A total of 50 mL of supernatant was collected at room temperature after this time for the isolation of EVs and secretome analysis.

### Extracellular vesicle separation, concentration and characterisation

Differential centrifugation and ultracentrifugation (CP80NX, Rotor P40ST, K factor at maximum speed of the rotor 139, HITACHI, Japan) were applied to isolate EVs. Briefly, 50 mL of supernatant was centrifuged in suitable tubes (Beckman, US Ref: 328874) for 10 min at 500 g to remove cells and large debris, then at 2500 g for 10 min to remove apoptotic bodies, then at 20,000 g for 20 min at 4 °C, and finally two 100,000 g centrifugation steps for 70 min at 4 °C each with a PBS wash in between. The pellet was resuspended in a total of 100 µl of protein lysis buffer (see below) except for an aliquot of each sample which was resuspended in 1 mL of PBS for nano-tracking analysis (NTA) which was used to determine the size and concentration of the isolated particles (Zetaview PMX110 Multiple Parameter Particle Tracking Analysis, ParticleMetrix, Meerbusch, Germany, in size mode) and for functional studies. If not used straightaway EV preparations were kept in 1.5 mL Eppendorf tubes at − 80 °C.

For NTA, vesicles were resuspended in 1 × PBS and diluted to the working range of the system (10^6^–10^10^ particles/mL). Videos were captured and analyzed with the ZetaView software (version 8.02.28, Meerbusch, Germany) using 11 camera positions, a 2-s video length, and a camera frame rate of 15 fps (for microparticles) or 30 fps (for exosomes) at 21 °C.

### Label-free quantitative proteomics and bioinformatic analysis

Sample preparation and protein digestion were performed as previously described^30,31^. Briefly, purified EVs or cells conditioned medium for secretome analysis were resuspended in Lysis Buffer containing 7 M urea, 2 M tiourea and 50 mM DTT. Protein extracts were precipitated with pre-chilled acetone, and pellets dissolved in the same Lysis Buffer. For protein digestion, protein extracts were reduced with DTT 10 mM at RT for 30 min. Cysteine residues were alkylated with iodoacetamide 30 mM at RT for 30 min. Protein enzymatic cleavage was carried out with trypsin (Promega, US; 1:20, w/w) at 37 °C for 16 h as previously described^32^. Purification and concentration of peptides was performed using C18 ZipTip Solid Phase Extraction (Millipore, Merck, Germany).

Peptides mixtures were separated by reverse phase chromatography using an Eksigent nanoLC ultra 2D pump fitted with a 75 mm ID column (Eksigent, USA, 0.075 × 150). Samples were first loaded for desalting and concentration into a 0.5 cm length 300 mm ID pre column packed with the same chemistry as the separating column. Mobile phases were 100% water, 0.1% formic acid (FA) (buffer A) and 100% Acetonitrile, 0.1%cFA (buffer B). Column gradient was developed in a 180 min step gradient from 2% B to 40% B. Column was equilibrated in 95% c B for 9 min and 5% c B for 14 min. During all process, precolumn was in line with column and flow maintained all along the gradient at 300 ml/min. Eluting peptides from the column were analyzed using a Sciex 5600 Triple-TOF system. Information data acquisition was acquired upon a survey scan performed in a mass range from 350 m/z up to 1250 m/z in a scan time of 250 ms. Top 30 peaks were selected for fragmentation. Minimum accumulation time for MS/MS was set to 100 ms giving a total cycle time of 3.8 s. Product ions were scanned in a mass range from 230 m/z up to 1500 m/z and excluded for further fragmentation during 15 s.

MS/MS data acquisition was performed using Analyst 1.7 (Sciex, US) and spectra ples were processed through Protein PilotTM Software 5.1 (Sciex, US) using ParagonTM Algorithm (v.4.0.0.0)^33^ for database search, Progroup™ for data grouping, and searched against the concatenated target-decoy UniProt human database. False discovery rate was performed using a non-lineal fitting method^34^ and displayed results were those reporting a 1% global false discovery rate or better. The peptide quantification was performed using the Progenesis LC–MS software (Waters, US). Using the accurate mass measurements from full survey scans in the TOF detector and the observed retention times, runs were aligned to compensate for between-run variations in our nanoLC separation system. To this end, all runs were aligned to a reference run automatically chosen by the software, and a master list of features considering m/z values and retention times was generated. The quality of these alignments was manually supervised with the help of quality scores provided by the software. The peptide identifications were exported from Protein Pilot and imported in Progenesis where they were matched to the respective features. Output data files were managed using Perseus software^35^ for statistical analysis and data visualization. Identification from the reverse database, common contaminants and proteins only identified through a modification peptide were removed. Label-free intensities were then logarithmized (base 2) and the samples were then grouped. At least two valid values across the three replicates were required for each identified protein. Following the Perseus analysis pipeline, empty values were imputed with random numbers from a normal distribution to simulate low abundance values below the detection limit of the instrument. For each condition, a two-sample t-test based on *p*-value < 0.05 was performed.

Proteomic files were deposited at the Proteome Xchange Consortium via the JPOST partner repository (https://repository.jpostdb.org) under the identifiers PXD042327 for ProteomeXchange and JPST002161 for jPOST.

### ELISA assay

For analysis of protein levels in serum we used commercially available ELISA kits according to the manufacturer's instructions (Human MMP3 ELISA ab100607, Abcam, Cambridge, UK).

### EVs uptake and cell motility tracking assays

To optimise the protocol for the treatment of cells with isolated EVs we studied the uptake and distribution of labelled EVs in vivo in control fibroblasts. EVs from these control cells were isolated as described above with some modifications according to the EVs dye manufacturers. Following the 10,000 g centrifugation step and before the 70 min 100,000 g ultracentrifugation step, Vybrant DIO (Thermofisher Scientific, US) at a final concentration of 3.75 µM was added to the EVs fraction. After washing with PBS and centrifuging again at 100,000 for 70 min the pellet with the labelled EVs was resuspended in 300 µl of PBS and stored at -80 C if not used straight away. For the treatment with EVs, 40,000 cells were seeded in 35 mm dishes (Idibi GmbH, Germany) and grown in complete DMEM medium with 10% FBS for 24 h. After this time, the medium was replaced with a medium complemented with EVs-free FBS (SIGMA, Germany) and the labelled EVs preparation was added and the cells were grown for a further 24 h. We tested different amounts of EVs based on existing literature^12^ and our data regarding the concentration of EVs in fibroblasts supernatants (determined by NTA) and their protein content (which was determined before proteomic analysis). Before imaging, 2 μg/ml of CellMask (Life Technologies; US) was added to the cells in EVs FBS-free medium and cells were incubated for 7 min at room temperature. After this time, the medium was removed and fresh EVs-free FBS medium was added.

To investigate the effect of EVs on cell motility, healthy control fibroblasts were treated with EVs derived from either their own supernatant or from supernatants from 2 of the patients with the *COL6A1* c.877 G > A; p. Gly 293Arg mutation and included in the proteomic study, one with an intermediate phenotype and another one with a milder Bethlem phenotype^3^. We seeded 170 cells/mL in 35 mm dishes (ibiTreat; Ibidi GmbH, Germany) which contain a 2-well insert with a defined 500 um cell-free gap and are specially designed for wound healing and migration experiments.

After 24 h we added 20,000 EVs per well at a concentration of 1,000 EVs/µl based on the optimization studies described above. After 24 h of treatment with EVs, dishes were washed with PBS and the silicone insert was removed to generate the wound. Cells were then labelled with 10 µM of Cell Tracker Deep Red (ThermoFisher, US) in serum-free medium for 50 min at 37 °C. After this time, the medium was withdrawn, and cells were washed twice with PBS. Finally, we added 3 mL of OptiMem (ThermoFisher, US) and cells were placed in the CO_2_ incubator attached to the confocal microscope for imaging. Cell movement was recorded using a TCS-SP8 (Leica Microsystems GmbH, Germany) confocal laser scanning microscope. Images were taken using a 10x (NA 0.75, dry) Plan-Apochromatic objective, an excitation wavelength at 633 nm and emission at 650–795 nm using a hybrid detector. The cell tracking was evaluated using 4D time-lapse. Projections were obtained from 20 serial optical sections (z-step = 4.5 µm). The stack of images was acquired every 10 min for 17 h. At least three independent experiments were performed and 1500–2300 cells for any condition were analyzed. The average speed of cells (m/s) in 3D was computed by tracking position vs. time using the built-in spots function of Imaris X64 v. 6.2.0 software (Bitplane, Zurich, Switzerland). This function was used to calculate centroids of fluorescent objects and to generate migratory tracks. Cell tracks were generated with an autoregressive motion algorithm and have been validated manually to eliminate all algorithm-generated errors. Average speed was assessed using the average track speed function in Imaris software and was calculated by the track length divided by the time between the first and last object in the track, and is expressed in µm/s. Directionality was assessed using the track straightness function in Imaris software that consists of the ratio between track displacement length (distance between first and last surface position) and track length (total length of displacements within the track), parameters also calculated. Data were analysed using PRISM 8 software.

### Supplementary Information

Supplementary Information 1.

Supplementary Information 2.

Supplementary Information 3.

Supplementary Video 1.

Supplementary Video 2.

Supplementary Video 3.

Supplementary Video 4.

Supplementary Video 5.

## Data Availability

Proteomic files were deposited at the Proteome Xchange^36^ Consortium via the JPOST partner repository (https://repository.jpostdb.org) under the identifiers PXD042327 for ProteomeXchange and JPST002161 for jPOST.

## References

[1] Lopez-Verrilli MA, Court FA (2013). Exosomes: Mediators of communication in eukaryotes. Biol. Res..

[2] Kalra H, Drummen GP, Mathivanan S (2016). Focus on extracellular vesicles: Introducing the next small big thing. Int. J. Mol. Sci..

[3] Natera-de Benito D (2021). Association of initial maximal motor ability with long-term functional outcome in patients with COL6-related dystrophies. Neurology.

[4] Lamandé SR, Bateman JF (2017). Collagen VI disorders: Insights on form and function in the extracellular matrix and beyond. Matrix Biol..

[5] Paco S (2015). Transcriptome analysis of Ullrich congenital muscular dystrophy fibroblasts reveals a disease extracellular matrix signature and key molecular regulators. PLoS ONE.

[6] Castroflorio E (2022). The capillary morphogenesis gene 2 triggers the intracellular hallmarks of collagen VI-related muscular dystrophy. Int. J. Mol. Sci..

[7] Keerthikumar S (2016). ExoCarta: A web-based compendium of exosomal cargo. J. Mol. Biol..

[8] Kalra H (2012). Vesiclepedia: a compendium for extracellular vesicles with continuous community annotation. PLoS Biol..

[9] Ji H (2008). Difference gel electrophoresis analysis of Ras-transformed fibroblast cell-derived exosomes. Electrophoresis.

[10] Luga V (2012). Exosomes mediate stromal mobilization of autocrine Wnt-PCP signalling in breast cancer cell migration. Cell.

[11] Anand S (2018). Arrestin-domain containing protein 1 (Arrdc1) regulates the protein cargo and release of extracellular vesicles. Proteomics.

[12] You Y (2019). Activated human astrocyte-derived extracellular vesicles modulate neuronal uptake, differentiation and firing. J. Extracell Vesicles.

[13] Nash LA (2017). Survival Motor neuron protein is released from cells in exosomes: A potential biomarker for spinal muscular atrophy. Sci. Rep..

[14] Jiang K (2019). Plasma exosomes from children with juvenile dermatomyositis are taken up by human aortic endothelial cells and are associated with altered gene expression in those cells. Pediatr. Rheumatol. Online J..

[15] Perez-Riverol Y (2019). The PRIDE database and related tools and resources in 2019: improving support for quantification data. Nucleic Acids Res..

[16] Szklarczyk D (2021). The STRING database in 2021: customizable protein-protein networks, and functional characterization of user-uploaded gene/measurement sets. Nucleic Acids Res..

[17] Théry C (2018). Minimal information for studies of extracellular vesicles 2018 (MISEV2018): A position statement of the International Society for Extracellular Vesicles and update of the MISEV2014 guidelines. J. Extracell Vesicles..

[18] Oliveros, J. C. Venny. An interactive tool for comparing lists with Venn's diagrams. (2007–2015). https://bioinfogp.cnb.csic.es/tools/venny/index.html.

[19] Zhou Y (2019). Metascape provides a biologist-oriented resource for the analysis of systems-level datasets. Nat. Commun..

[20] Keerthikumar S (2016). A catalogue of human secreted proteins and its implications. J. AIMS Biophys..

[21] Loredo Martinez M (2020). Nonimmune mechanisms in idiopathic inflammatory myopathies. Curr. Opin. Rheumatol..

[22] Pasetto L (2021). Decoding distinctive features of plasma extracellular vesicles in amyotrophic lateral sclerosis. Mol. Neurodegener.

[23] Paco S (2013). Gene expression profiling identifies molecular pathways associated with collagen VI deficiency and provides novel therapeutic targets. PLoS ONE.

[24] Tawalbeh S (2020). Comparison of serum pharmacodynamic biomarkers in prednisone-versus deflazacort-treated duchenne muscular dystrophy boys. J. Pers. Med..

[25] Sung BH, Parent CA, Weaver AM (2021). Extracellular vesicles: Critical players during cell migration. Dev. Cell.

[26] Maziveyi M (2019). Exosomes from nischarin-expressing cells reduce breast cancer cell motility and tumor growth. Cancer Res..

[27] Margolis L, Sadovsky Y (2019). The biology of extracellular vesicles: The known unknowns. PLoS Biol..

[28] Chiang CY, Chen C (2019). Toward characterizing extracellular vesicles at a single-particle level. J. Biomed. Sci..

[29] López-Márquez A (2022). CRISPR/Cas9-mediated allele-specific disruption of a dominant *COL6A1* pathogenic variant improves collagen VI network in patient fibroblasts. Int. J. Mol. Sci..

[30] Zelaya MV, Pérez-Valderrama E, de Morentin XM, Tuñon T, Ferrer I, Luquin MR, Fernandez-Irigoyen J, Santamaría E (2015). Olfactory bulb proteome dynamics during the progression of sporadic Alzheimer's disease: identification of common and distinct olfactory targets across Alzheimer-related co-pathologies. Oncotarget.

[31] Lachen-Montes M, Zelaya MV, Segura V, Fernández-Irigoyen J, Santamaría E (2017). Progressive modulation of the human olfactory bulb transcriptome during Alzheimer´s disease evolution: Novel insights into the olfactory signaling across proteinopathies. Oncotarget.

[32] Shevchenko A, Tomas H, Havlis J, Olsen JV, Mann M (2006). In-gel digestion for mass spectrometric characterization of proteins and proteomes. Nat. Protoc..

[33] Shilov IV, Seymour SL, Patel AA, Loboda A, Tang WH, Keating SP, Hunter CL, Nuwaysir LM, Schaeffer DA (2007). The Paragon Algorithm, a next generation search engine that uses sequence temperature values and feature probabilities to identify peptides from tandem mass spectra. Mol. Cell Proteomics.

[34] Tang WH, Shilov IV, Seymour SL (2008). Nonlinear fitting method for determining local false discovery rates from decoy database searches. J. Proteome Res..

[35] Tyanova S, Temu T, Cox J (2016). The MaxQuant computational platform for mass spectrometry-based shotgun proteomics. Nat. Protoc..

[36] Vizcaíno JA, Deutsch EW, Wang R, Csordas A, Reisinger F, Ríos D, Dianes JA, Sun Z, Farrah T, Bandeira N, Binz PA, Xenarios I, Eisenacher M, Mayer G, Gatto L, Campos A, Chalkley RJ, Kraus HJ, Albar JP, Martinez-Bartolomé S, Apweiler R, Omenn GS, Martens L, Jones AR, Hermjakob H (2014). ProteomeXchange provides globally coordinated proteomics data submission and dissemination. Nat. Biotechnol..

