# Supplementary figures and images for: Proteomic and functional characterisation of extracellular vesicles from collagen VI deficient human fibroblasts reveals a role in cell motility
Source: Sci Rep. 2023 Sep 5;13:14622. doi: 10.1038/s41598-023-41632-1 (PMC10480450; doi:10.1038/s41598-023-41632-1)

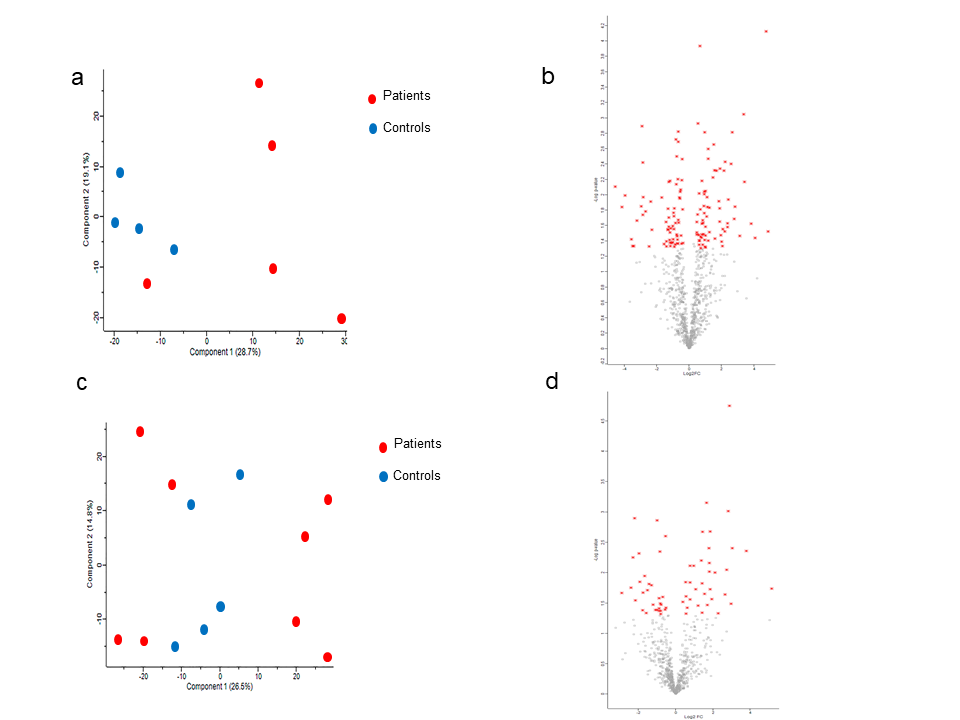

Supplement: Supplementary file 1 — Supplementary Information 1. [file 41598_2023_41632_MOESM1_ESM.tif]
