# Supplementary material for: Proteomic and functional characterisation of extracellular vesicles from collagen VI deficient human fibroblasts reveals a role in cell motility
Source: Sci Rep. 2023 Sep 5;13:14622. doi: 10.1038/s41598-023-41632-1 (PMC10480450; doi:10.1038/s41598-023-41632-1)
Supplement: Supplementary file 3 — Supplementary Information 3. [file 41598_2023_41632_MOESM3_ESM.docx]

**Supplementary Material**

Supp. Fig 1. Principal component analysis (PCA) based on protein abundance levels across runs, separating control and patient samples corresponding to the analysis of the EVs (a) and secretome (c). Some samples were eliminated from the final analysis due to the erratic behavior during nanoHPLC-MS/MS phase. Volcano plots representing the fold-change versus significance level of the different proteins obtained in the analysis of the EVs (b) and the secretome (d).

Supp.Table 1. Gene/protein datasets

Supp. Video 1 Representative in vivo imaging showing the incorporation of labelled EVs in control fibroblasts. In blue: cell nuclei, in red: cell membrane, in green: internalized EVs.

Supp. Video 2 Cell tracking experiments (Time lapse, 17 hours). Control fibroblasts untreated.

Supp. Video 3. Cell tracking experiments (Time lapse, 17 hours). Control fibroblasts treated with EVs derived from control fibroblasts.

Supp. Video 4. Cell tracking experiments. (Time lapse, 17 hours) Control fibroblasts treated with EVs derived from a COL6-RD muscular dystrophy patient with an intermediate phenotype.

Supp. Video 5. Cell tracking experiments (Time lapse, 17 hours). Control fibroblasts treated with EVs from a COL6-RD muscular dystrophy patient with a mild phenotype.
